# Level of 25-hydroxyvitamin D and vitamin D receptor in diabetic foot ulcer and factor associated with diabetic foot ulcers

**DOI:** 10.1186/s13098-023-01002-3

**Published:** 2023-02-24

**Authors:** Ying Tang, Yixuan Huang, Li Luo, Murong Xu, Datong Deng, Zhaohui Fang, Xiaotong Zhao, Mingwei Chen

**Affiliations:** 1 Department of Endocrinology, The First Affiliated Hospital of Anhui Medical University, Hefei, Anhui People’s Republic of China; 2 Department of Endocrinology, The First Affiliated Hospital of Anhui University of Traditional Chinese Medicine, Hefei, Anhui People’s Republic of China

**Keywords:** Diabetic foot ulcer, Diabetic foot osteomyelitis, 25-Hydroxyvitamin D, Type 2 diabetes mellitus, Vitamin D receptor

## Abstract

**Background:**

At present, there is no clinical study to elucidate the correlation between vitamin D deficiency and the incidence of diabetic foot osteomyelitis (DFO).This study aims to clarify levels of 25-hydroxyvitamin D [25(OH)VD] in peripheral blood and vitamin D receptor (VDR) expression in wound margin tissues (T-VDR) of patients with type 2 diabetes mellitus (T2DM) with diabetic foot ulcer (DFU) and DFO, and to determine its correlation with treatment outcomes of DFU and DFO, and and its value as a potential biomarker for the diagnosis of DFU and DFO.

**Methods:**

156 T2DM patients with DFU (DFU group), 100 T2DM patients without DFU (T2DM group), and 100 healthy controls (NC group). The DFU group patients were subdivided into DFO (n = 80) and NDFO groups (n = 76). The level of serum 25(OH)VD was measured via chemiluminescence immunoassay, and T-VDR expression level was determined by quantitative real-time PCR.

**Results:**

The levels of serum 25(OH)VD in the DFU group were significantly lower than the T2DM group [(10.3 (5.8, 18.7) vs 15.7 (8.6, 24.6) ng/mL, *P* = 0.002)]. Similarly, the levels of serum 25(OH)VD and T-VDR expression in the DFO group were statistically lower than the NDFO group [9.2 (5.2, 20.5) vs 12.8 (6.9, 22.1) ng/mL, *P* = 0.006)], [1.96 (0.61, 3.97) vs 3.11 (1.36, 5.11), *P* = 0.004)], respectively. Furthermore, the levels of serum 25(OH)VD and T-VDR expression in DFU patients were positively correlated with the ulcer healing rate of foot ulcer after 8 weeks of treatment ( *P* = 0.031, *P* = 0.016, respectively). Multivariate logistic regression analysis showed that low level of serum 25(OH)VD was an independent risk factor for DFU and DFO (OR_DFU_ = 2.42, OR_DFO_ = 3.05, *P* = 0.008, 0.001, respectively), and decreased T-VDR expression level was an independent risk factor for DFO (OR = 2.83, *P* = 0.004). Meanwhile, the ROC curve analysis indicated that the AUC of serum 25(OH)VD level for the diagnosis of DFU and DFO was 0.821 (95% CI, 0.754–0.886, *P* < 0.001) and 0.786 (95%CI, 0.643–0.867, *P* < 0.001), respectively. When establishing a diagnosis of DFO, the AUC of T-VDR expression level was 0.703 (95%CI: 0.618–0.853, *P* < 0.001).

**Conclusions:**

The levels of serum 25(OH)VD and T-VDR expression in DFU and DFO decreased. Serum 25(OH)VD and T-VDR are potentially valuable biomarkers for diagnosis and prognosis of DFU and DFO.

.

**Supplementary Information:**

The online version contains supplementary material available at 10.1186/s13098-023-01002-3.

## Introduction

Diabetic foot ulcer (DFU) is a severe chronic complication of diabetes mellitus (DM) and is associated with a remarkably high treatment cost. Approximately 15% of patients with DM are reported to present with DFU at some time in their lives [1]. The presence of DFU in combination with an infection can impede wound healing, and then progress to diabetic foot osteomyelitis (DFO), leading to increased mortality, decreased quality of life, and increased risk of lower limb amputation [2].

Vitamin D is a type of steroid prohormone. The vitamin D receptor (VDR) exists in nearly all tissues in the human body, such as intestines, bones, parathyroid glands, kidneys, reproductive system, immune cells and is particularly abundant in the skin [3]. It is a member of the steroid hormone receptor superfamily and can regulate the expression of corresponding target genes after being activated. In vitro studies have revealed that a high concentration of vitamin D can upregulate the VDR expression. Several types of vitamin D are currently known, among which vitamin D3 has the highest activity. Vitamin D3 undergoes two steps of hydroxylation and is converted into 1,25(OH)2D3; this compound then combines with VDR to exert physiological effects. Although 1,25(OH)2D3 was considered the active form of vitamin D, its level in serum has not been correlated with the systemic levels of vitamin D. Conversely, 25-hydroxyvitamin D (25(OH)VD) level is known to reflect the systemic vitamin D level [4].

According to the previous survey [5], the concentration of 25(OH)VD varies from population to population. Summer is usually higher than winter. The concentration of 25(OH)VD varies according to different geographical locations, latitude, eating habits and environmental climate. However, the most likely reason for the poor status of vitamin D is the insufficient endogenous synthesis caused by insufficient ultraviolet radiation. Research shows that the prevalence of vitamin D insufficiency and deficiency is very high in the general population of the world, and the situation of vitamin D deficiency in DFU patients is more obvious [6]. Some studies have reported that low level of serum 25(OH)VD can increase the risk of DFU and DFU infection [7, 8], and are related to an increase in the risk of amputation or mortality [9]. Additionally, vitamin D has been involved in regulating the healing process of chronic wounds [10], Vitamin D supplementation can promote wound healing in cases of DFU [11]. However, some studies have not reported a correlation between vitamin D deficiency and the onset of DFU [9, 12]; furthermore, the levels of serum 25(OH)VD were reported to be significantly increased in patients with chronic active DFU [13]. Owing to the complexity of DFU pathogenesis, further investigation of the correlation between vitamin D insufficiency and DFU pathogenesis and its influencing factors is of paramount importance. Additionally, to the best of our knowledge, no clinical studies have attempted to elucidate the correlation between vitamin D deficiency and the incidence of DFO.

Therefore, in the present study, we aimed to further explore the levels of 25(OH)VD in peripheral blood and VDR expression in wound margin tissues of patients with DFU and DFO, and identify its correlation with treatment outcomes of DFU and DFO, including foot ulcer recurrence, healing, and amputation, and its value as a potential biomarker for the diagnosis of DFU and DFO.

## Material and methods

### Study subjects

**Fig. 1:**
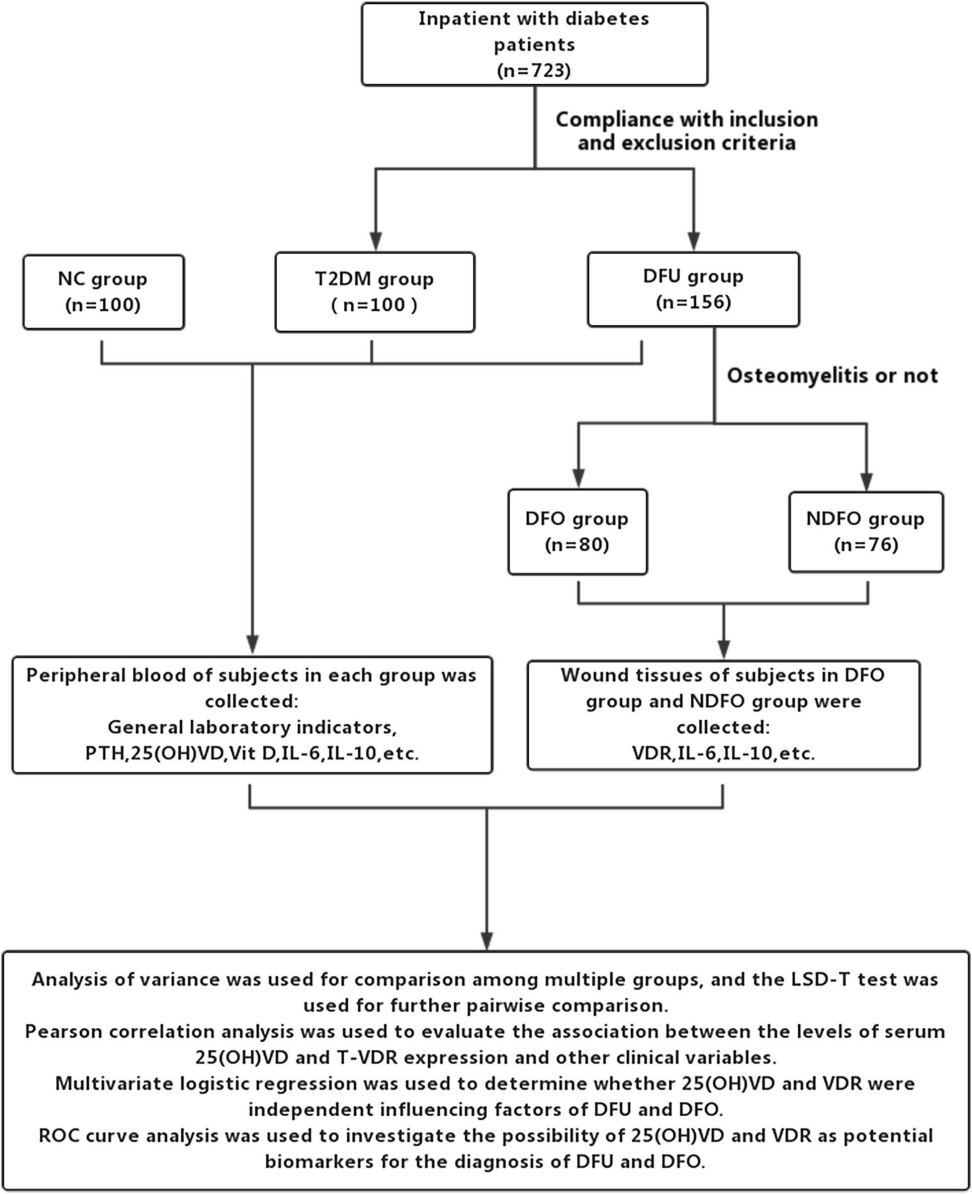
Research design drawing. *NC* normal control group, *T2DM* type 2 diabetes mellitus group, *DFU* diabetic foot ulcer, *DFO* diabetic foot osteomyelitis, *NDFO* diabetic foot ulcer without osteomyelitis, *PTH* parathyroid hormone,*25(OH)VD* 25-hydroxyvitamin D, *Vit D* vitamin D, *IL-6* interleukin-6, *IL-10* interleukin-10,*VDR* vitamin D receptor

We included 723 diabetes patients hospitalized in the Department of Endocrinology of of the First Affiliated Hospital of Anhui Medical University from January 2019 to January 2021, of which 156 DFU patients were selected as the DFU group. All DFU patients met the following inclusion criteria: (1) the ulcer duration was four weeks or longer; (2) the ulcer area was 2–20 cm^2^ and Wagner grade 2–4; (3) the ankle-brachial index (ABI) was 0.4–0.9; and (4) being diagnosed as type 2 diabetes mellitus (T2DM). Diagnose DFU with osteomyelitis based on medical history, physical signs (especially probe examination), and imaging examination results (X-ray or magnetic resonance imaging [MRI]). Based on this, 156 patients with DFU were labeled into two groups: osteomyelitis group (DFO group, n = 80) and non-osteomyelitis group (NDFO group, n = 76). Meanwhile, we selected 100 T2DM patients from the remaining 567 inpatients with diabetes as the control group of DFU (T2DM group). All patients in the T2DM group were hospitalized for further examination and treatment due to polyuria, polydipsia, polyphagia, weight loss, or abnormal increase of fasting blood glucose or glycosylated hemoglobin A1c (HbA1c) found in the physical examination, and there were no foot ulcers, diabetes lower limb atherosclerotic disease, and diabetes peripheral neuropathy. Besides, another 100 healthy individuals who underwent physical examination at the health management center of our hospital during the study period were selected as the normal control group (NC group). Subjects in the NC group was received 75 g oral glucose tolerance test (OGTT). Normally, a normal test result requires fasting blood glucose (FPG) level is lower than 6.1 mmol/L, and the blood glucose level 2 h after the glucose load is lower than 7.8 mmol/L. Significantly, exclusion criteria for the subjects were with being bedridden for a long time, acute complications related to diabetes, severe heart, liver, and kidney dysfunction, cancerous ulcer wound, parathyroid disease, autoimmune diseases, taking any drugs that affect serum 25 (OH) VD level, such as calcium, vitamin D, oral contraceptives and glucocorticoids, etc., severe septicemia. The above research design is shown in Fig. 1. The study was approved by the medical ethics committee of the First Affiliated Hospital of Anhui Medical University (Ethical batch number P 2018-11-16), and written informed consent was obtained from all subjects.

## Study methods

### Treatment process of DFU

All patients with DFU underwent wound debridement after admission. In the process of debridement, a skilled surgeon used tissue scissors to cut the full-thickness skin tissue within 0.5 cm of the wound edge according to the sampling protocol. All patients with DFU received routine systemic treatment, which included anti-infection treatment, blood pressure regulation, blood glucose reduction, correction of hypoproteinemia, nerve-nutrition treatment, improvement of blood supply to lower extremity wounds, and wound debridement. The abovementioned procedures were performed to remove blackened necrotic soft tissue and bone tissue. Decompression and continuous negative pressure wound treatment (NPWT) were performed on a case-by-case basis for the patients with DFU. Amputation was decided by a multidisciplinary team (MDT) of diabetic foot after consultation; the factors that affected the final decision were changes in the patient’s condition during the follow-up period; in addition, complete epithelial healing of the wound was observed after 8 weeks of treatment [14]. Briefly, if the wound epithelium is completely healed within 8 weeks or less, the wound is judged to be healed. If the wound epithelium is not completely healed for more than 8 weeks, the wound is judged to be not healed.

### Collection of general information

We measured the systolic blood pressure (SBP) and diastolic blood pressure (DBP) of the subjects and recorded the season at the time of enrollment and the mean duration of exposure to sunshine in the recent 3 months. Data related to new or recurrent foot ulcers, smoking habits, and alcohol consumption were recorded. Regular smoking was defined as smoking at least 1 cigarette a day for a mean duration of > 6 months. Chronic alcohol consumption was defined as consuming more than 40 g of ethanol per day for men and 20 g per day for women for more than 5 consecutive years. The severity of DFU infection (mild, moderate, and severe) was assessed according to the recommendations of the Infectious Disease Society of America (IDSA) [15]. The ulcer area was measured via digital photography combined with Image J Medical Image analysis software (Image J-IJ133-JDK15, National Institutes of Health, Bethesda, USA). ABI was measured using a Doppler blood flow detector (DPL-03, Hangzhou Yuanxiang Medical, China). The transcutaneous oxygen pressure (TcPO_2_) in the vicinity of the ulcer was measured using a TcPO_2_ monitor (TCM 400, Denmark).

### Detection of observation indices

After fasting for 10 h, we collected blood from the median vein of the elbow in the fasting state from 8:00 am to 8:30 am of the next day to determine liver and kidney function, blood glucose levels, blood lipid composition, HbA1c levels, white blood cell (WBC) count, c-reactive protein (CRP), erythrocyte sedimentation rate (ESR), serum 25(OH)VD, serum-free calcium, IL-6, IL-10, and parathyroid hormone (PTH), among other indicators (See Additional file 2: Table S1 for detailed determination method). Estimated glomerular filtration rate (eGFR) was used to evaluate renal function. eGFR (ml/min / 1.73 m^2^) = 186 × (Scr ^−1.154^) × (age^−0.203^) × [0.742 (female)] [16]. Vitamin D nutritional status was categorized as deficiency, insufficiency, and adequacy when vitamin D levels were < 20 ng/mL (< 50 nmol/L), 20–30 ng/mL (50–75 nmol/L), and > 30 ng/mL (> 75 nmol/L), respectively [17]. Additionally, quantitative real-time polymerase chain reaction (qRT-PCR) was used to determine the levels of VDR mRNA (T-VDR), IL-6 mRNA (T-IL-6), and IL-10 mRNA (T-IL-10) in wound margin tissues. RNA was extracted from 50 mg of wound edge tissue according to the manufacturer instructions of the miRcute miRNA Extraction and Isolation Kit (Tiangen Biochemical Technology Co., LTD, Beijing, China). The cDNA was then synthesized according to the manufacturer instructions of the miRcute miRNA cDNA Synthesis Kit (Tiangen Biochemical Technology Co., LTD., Beijing, China). Finally, qRT-PCR was performed according to the manufacturer’s instructions of the miRcute miRNA fluorescence quantitative detection kit (See Additional file 3: Table S2 for specific primers and reaction conditions). The relative levels of T-VDR, T-IL-6, and T-IL-10 were calculated using the 2^−△△Ct^ method with GAPDH as the internal reference.

### Statistical analyses

Statistical software SPSS22.0 (Chicago, IL, USA) was used for data analysis. Measurement data with normal distribution were expressed as mean ± standard deviation, and measurement data with non-normal distribution were expressed as median (interquartile range) [M (P_25_, P_75_)]. The Student’s t-test and the Mann–Whitney U test were used to compare normally and non-normally distributed data. Analysis of variance was used for comparison among multiple groups, and the LSD-T test was used for further pairwise comparison. Enumeration data were expressed as a percentage and the χ^2^ test was performed. Pearson correlation analysis was used to evaluate the association between the levels of serum 25(OH)VD and T-VDR expression and other clinical variables. Multivariate logistic regression was used to determine the risk factors of DFU and DFO. Receiver operating characteristic (ROC) curve analysis was used to investigate the potential biomarkers for the diagnosis of DFU and DFO. All tests were bilateral, and a *P*-value of < 0.05 indicated statistical significance.

## Results

### Comparisons in clinical parameters of NC group, T2DM group, DFU group

No significant differences were observed in terms of sex, age, smoking habits, alcohol consumption, the proportion of subjects enrolled in winter, mean sunshine duration per day in the recent 3 months, SBP, DBP, TCH, LDL-C, PTH, IL-10 levels in peripheral blood (P-IL-10), and serum ionized calcium levels among the NC, T2DM, and DFU groups (*P* > 0.05). On the one hand, compared with the NC group, the levels of FPG, HbA1c and TG in the T2DM and DFU groups were significantly increased, and the proportion of vitamin D deficiency and insufficiency were significantly increased (*P* < 0.05). Otherwise, the levels of HDL-C and serum 25(OH)VD were decreased significantly (*P* < 0.05). On the other hand, compared with the T2DM group, the duration of diabetes, FPG, HbA1c, TG, CRP, IL-6 levels in peripheral blood (P-IL-6), WBC, and ESR levels appeared to increase significantly in the DFU group (*P* < 0.05), while the mean sunshine duration per day in the recent 3 months, eGFR, serum albumin, hemoglobin, serum 25(OH)VD, TcPO2, ABI levels significantly decreased (*P* < 0.05). In particularly, serum 25(OH)VD levels in the DFU group were statistically lower than the T2DM group [10.3 (5.8, 18.7) vs 15. 7 (8.6, 24.6) ng/mL, *P* = 0.002)] (Table 1). Additionally, in the present study, with the increase of Wagner grade, the detection rate of vitamin D deficiency and insufficiency in DFU increased significantly (χ^2^ = 40.31, *P* < 0.001). Similarly, with the increase of infection severity, the detection rate of vitamin D deficiency and insufficiency in DFU also increased correspondingly (χ^2^ = 23.86, *P* < 0.001). In particular, the proportion of vitamin D deficiency and insufficiency in Wagner IV and severely infected DFUs was 100% (Additional file 4: Table S3).

**Table 1:** Comparisons in clinical parameters among NC group, T2DM group, and DFU group [n (%), ( ± s), M (P25, P75)]

Variable	NC group (n = 100)	T2DM group (n = 100)	DFU group (n = 156)	*P* value
Sex (male/female)	100(57/43)	100(54/46)	156 (88/68)	0.899
Age (year)	55.1 ± 11.9	54.9 ± 12.3	55.6 ± 11.2	0.581
Duration of diabetes (year)	–	0.4 ± 0.2	12.1 ± 6.4^d^	< 0.001
Duration of ulcers (week)	–	–	7.2 ± 2.6	–
Smoking habits	44 (44.0)	48 (48.0)	80 (51.3)	0.522
Alcohol consumption	23 (23.0)	26 (26.0)	43 (27.6)	0.717
Mean sunshine time per day (h)	4.1 (1.9, 6.3)	3.9 (1.7, 6.1)	2.8 (1.2, 5.7)^bd^	0.002
Winter enrollment	39 (39.0)	40 (40.0)	69 (44.2)	0.661
Wagner grade (2/3/4)	–	–	20/108/28	–
Severity of infection (mild/ moderate/ severe)	–	–	20/94/42	–
First episode/ recurrence of foot ulcer	–	–	105/51	–
SBP (mm Hg)	122 ± 11	127 ± 13	132 ± 14	0.082
DBP (mm Hg)	74 ± 12	78 ± 13	82 ± 14	0.078
FPG (mmol/L)	4.9 ± 0.5	9.8 ± 2.4^b^	12.3 ± 2.7^bd^	< 0.001
HbA1c (%)	5.2 ± 0.4	8.5 ± 1.7^b^	9.3 ± 1.6^bc^	< 0.001
TG (mmol/L)	1.4 ± 0.6	1.8 ± 0.6^b^	2.2 ± 0.7^bc^	0.021
TCH (mmol/L)	4.2 ± 0.7	4.9 ± 0.6	4.6 ± 0.7	0.558
LDL-C (mmol/L)	2.5 ± 0.3	2.9 ± 0.4	2.8 ± 0.5	0.318
HDL-C (mmol/L)	1.5 ± 0.2	1.2 ± 0.3^a^	0.9 ± 0.3^bc^	< 0.001
PTH (pg/mL)	14.5 ± 4.2	15.1 ± 5.3	17.8 (10.5,42.4)	0.274
Serum ionic calcium (mmol/L)	0.92 ± 0.22	0.89 ± 0.19	0.86 ± 0.16	0.652
eGFR (ml/min/1.73m^2^)	104.4 ± 16.5	98.4 ± 10.2	75.7 ± 15.6^bc^	< 0.001
ALB (g/L)	45.2 ± 3.2	44.2 ± 3.5	37.2 ± 4.8^bc^	0.014
Hb (g /L)	134.4 ± 8.4	129.4 ± 10.2	113.7 ± 12.6^bc^	0.028
TcPO2 (mmHg)	76.4 ± 7.2	70.5 ± 8.9	48.5 ± 10.2^bd^	< 0.001
ABI	1.15 ± 0.14	1.04 ± 0.19	0.74 ± 0.29^bd^	< 0.001
CRP ( mg/L)	6.5 ± 0.9	7.1 ± 1.0	46.8 (27.5, 92.4)^bd^	< 0.001
WBC (× 10^9^)	4.3 ± 0.5	4.7 ± 0.6	11.5 ± 4.3^bd^	< 0.001
ESR (mm/h)	12.6 ± 1.3	12.8 ± 2.2	33.8 (25.9, 89.5)^bd^	< 0.001
25(OH)VD (ng/mL)	23.8 (15.7,33.7)	15. 7 (8.6,24.6)^a^	10.3(5.8, 18.7) ^bc^	< 0.001
Vit D status				< 0.001
Deficiency	28 (28.0)	54 (54.0)	112 (71.8)^bc^	
Insufficiency	52 (52.0)	36 (36.0)	36 (23.1)^bc^	
Sufficiency	20 (20.0)	10 (10.0)	8 (5.1)^bc^	
P-IL-6 (pg/ml)	14.1 ± 4.4	21.4 ± 8.3	48.4 ± 12.5^bd^	< 0.001
P-IL-10 (ng/L)	4.6 ± 1.1	4.0 ± 1.3	3.5 ± 1.5	0.105
T-IL-6	–	–	3.58 (1.87, 6.25)	–
T-IL-10	–	–	0.59 (0.14, 1.26)	–
T-VDR	–	–	2.68 (0.65, 5.26)	–

Data are presented mean ± standard deviations or numbers (%) or median with IQR; Differences among three groups analyzed using one-way analysis of variance or *x*^2^ test, and least-significant difference (LSD) analysis was used for comparison between the two groups. versus NC group, ^a^*P* < 0.05, ^b^*P* < 0.01; versus T2DM group, ^c^*P* < 0.05, ^d^*P* < 0.01

*NC* normal control group, *T2DM* type 2 diabetes group, *DFU* diabetic foot ulcer group, *SBP* systolic blood pressure, *DBP* diastolic blood pressure, *FPG* fasting plasma glucose, *HbA1c* glycated hemoglobin A1c, *TG* triacylglycerol, *TCH* total cholesterol, *LDL-C* low-density lipoprotein cholesterol, *HDL-C* high-density lipoprotein cholesterol, *PTH* parathyroid hormone, *eGFR* estimated glomerular filtration rate, *TcPO2* transcutaneous oxygen pressure, *ABI* ankle brachial index, *CRP* C-reactive protein, *WBC* white blood cell, *ESR* erythrocyte sedimentation rate, *25(OH)VD* 25-hydroxyvitamin D, *Vit D* vitamin D, *IL* interleukin, *P-IL-6* IL-6 level in peripheral blood, *P-IL-10* IL-10 level in peripheral blood, *T-IL-6* IL-6 mRNA expression in wound margin tissue, *T-IL-10* IL-10 mRNA expression in wound margin tissue, *T-VDR* vitamin D receptor mRNA expression in wound margin tissue

### Comparisons in clinical parameters between NDFO group and DFO group

Compared with the NDFO group, the duration of diabetes, the duration of foot ulcer, Wagner grade, infection severity, the proportion of foot ulcer recurrence, the proportion of drug-resistant bacteria detected in the wound, the proportion of vitamin D deficiency, FPG, HbA1c, CRP, WBC, ESR, P-IL-6, and T-IL-6 in the DFO group were significantly increased (*P* < 0.05). However, the mean sunshine duration per day in the recent 3 months, eGFR, serum ALB, Hb, serum 25(OH)VD level, P-IL-10 level, TcPO2, ABI, T-IL-10, and T-VDR expression level appeared to decrease significantly (*P* < 0.05). In addition, the levels of serum 25(OH)VD and T-VDR expression in the DFO group were statistically lower than the NDFO group [9.2 (5.2, 20.5) vs 12.8 (6.9, 22.1) ng/mL, *P* = 0.006)], [1.96 (0.61, 3.97) vs 3.11 (1.36, 5.11), *P* = 0.004)], respectively. From the analysis above, there were no significant differences between the NDFO group and the DFO group in terms of other clinical parameters (*P* > 0.05) (Table 2).

**Table 2:** Comparisons in clinical parameters between NDFO group and DFO group [n (%), ( ± s), M (P25, P75)]

Variable	NDFO group (n = 72)	DFO group (n = 84)	*P* value
Sex (male/female)	72(42/30)	84(46/38)	0.654
Age (year)	55.2 ± 11.6	55.8 ± 10.9	0.451
Duration of diabetes (year)	10.8 ± 5.7	13.9 ± 6.8	0.014
Duration of ulcers (week)	5.1 (4.0, 9.3)	6.8 (4.8, 16.2)	0.003
Area of DFU (cm^2^)	11.5 ± 4.3	10.7 ± 3.9	0.271
Smoking habits	38 (52.8)	42 (50.0)	0.729
Alcohol consumption	23 (31.9)	20 (23.8)	0.854
Mean sunshine time per day (h)	3.2 (1.2, 5.8)	2.1 (1.1, 4.9)	0.025
Winter enrollment	30 (41.7)	39 (46.4)	0.551
Wagner grade			< 0.001
II	20 (27.8)	0 (0.00)	
III	47 (65.3)	61 (72.6)	
IV	5 (6.9)	23 (27.4)	
Severity of infection			< 0.001
Mild	20 (27.8)	0 (0.00)	
Moderate	40 (55.6)	54 (64.3)	
Severe	12 (6.6)	30 (35.7)	
Recurrent DFU	17 (23.6)	34 (40.5)	0.033
Detection rate of drug -resistant bacteria	17 (23.6)	34 (40.5)	0.025
SBP (mm Hg)	121 ± 13	125 ± 15	0.487
DBP (mm Hg)	81 ± 14	83 ± 15	0.679
FPG (mmol/L)	11.2 ± 2.5	12.8 ± 2.8	0.032
HbA1c(%)	8.7 ± 1.5	9.5 ± 1.8	0.024
TG (mmol/L)	1.8 ± 0.8	2.4 ± 0.9	0.103
TCH (mmol/L)	4.7 ± 0.8	4.5 ± 0.7	0.594
LDL-C (mmol/L)	2.7 ± 0.5	2.9 ± 0.6	0.626
HDL-C (mmol/L)	1.0 ± 0.2	0.9 ± 0.3	0.753
PTH (pg/mL)	16.4 (9.7, 28.6)	18.1 (10.9, 39.2)	0.102
Serum ionic calcium (mmol/L)	0.85 ± 0.15	0.87 ± 0.17	0.285
eGFR (ml/min/1.73m^2^)	83.7 ± 11.9	71.5 ± 12.6	0.042
ALB (g/L)	40.7 ± 8.9	35.7 ± 6.5	0.038
Hb (g /L)	122.8 ± 9.3	104.8 ± 8.6	0.046
TcPO2 (mm Hg)	52.8 ± 8.5	39.1 ± 10.1	0.013
ABI	0.82 ± 0.21	0.65 ± 0.14	0.026
CRP ( mg/dL)	31.7 ± 13.7	62.2 ± 18.5	< 0.001
WBC (× 10^9^)	10.2 ± 3.6	12.9 ± 3.8	0.002
ESR (mm/h)	36.7 ± 12.8	67.9 ± 17.2	< 0.001
25(OH)VD (ng/mL)	12.8 (6.9, 22.1)	9.2 (5.2, 20.5)	0.006
VitD status			0.007
Deficiency	43 (59.7)	69 (82.1)	
Insufficiency	23 (31.9)	13 (15.5)	
Sufficiency	6 (8.4)	2 (2.4)	
P-IL-6 (pg/ml)	39.2 ± 12.8	51.7 ± 14.5	0.009
P-IL-10 (ng/L)	4.7 ± 1.6	3.1 ± 1.2	0.036
T-IL-6	2.93 (1.54, 4.82)	3.82 (2.07, 6.23)	0.013
T-IL-10	0.72 (0.31, 1.32)	0.48 (0.12, 0.95)	0.036
T-VDR	3.11 (1.36, 5.11)	1.96 (0.61–3.97)	0.004

Data are presented mean ± standard deviations or numbers (%) or median with IQR; Differences between two groups analyzed using t test or nonparametric test (Mann Whitney U)

*DFU* diabetic foot ulcer, *NDFO* diabetic foot ulcer without osteomyelitis, *DFO* diabetic foot osteomyelitis, *SBP* systolic blood pressure, *DBP*:diastolic blood pressure, *FPG* fasting plasma glucose, *HbA1c* glycated hemoglobin *A1c*, *TG*:triacylglycerol, *TCH*:total cholesterol, *LDL-C*:low-density lipoprotein cholesterol, *HDL-C*:high-density lipoprotein cholesterol, *PTH* parathyroid hormone, *eGFR* estimated glomerular filtration rate, *ALB* serum albumin, *Hb* haemoglobin, *TcPO2* transcutaneous oxygen pressure, *ABI* ankle brachial index, *CRP* C-reactive protein, *WBC* white blood cell, *ESR* erythrocyte sedimentation rate;25(OH)VD 25-hydroxyvitamin D, *Vit D* Vitamin D, *IL* interleukin, *P-IL-6* IL-6 level in peripheral blood, *P-IL-10* IL-10 level in peripheral blood, *T-IL-6* IL-6 mRNA expression in wound margin tissue, *T-IL-10* IL-10 mRNA expression in wound margin tissue, *T-VDR* vitamin D receptor mRNA expression in wound margin tissue

### Relationship between the levels of serum 25(OH)VD and T-VDR expression and the clinical features of DFU and DFO patients

In order to further study the clinical significance of changes in the levels of serum 25(OH)VD and T-VDR expression, median values of the levels of serum 25(OH)VD and T-VDR expression of patients with DFU and DFO were used as the cutoff points for grouping (The cut-off point of the levels of serum 25(OH)VD and T-VDR expression for grouping were 10.3 ng/mL and 2.68, respectively), and furthermore, the low-expression group (lower than the cut-off point) and high-expression group (higher than or equal to the cut-off point). Based on the comparison of the clinical characteristics of the two groups of DFU, the levels of serum 25(OH)VD and T-VDR expression were negatively correlated with the duration of foot ulcer disease, Wagner grade, the severity of wound infection, the detection rate of drug-resistant bacteria, the recurrence rate of foot ulcer, and amputation rate of foot ulcer (*P* < 0.05), and positively correlated with the healing rate of foot ulcer after 8 weeks (*P* < 0.05). No correlation was observed between the levels of serum 25(OH)VD and T-VDR expression and other clinical features of foot ulcer (Tables 3, 4). Additionally, Research data also showed the relationship between the levels of T-VDR expression and the clinical features of DFO is similar to that found in foot ulcer (Table 5).

**Table 3:** Relationship between serum 25(OH)VD levels and the clinical features of DFU patients [( ± s), n (%)]

	High level group (n = 58)	Low level group (n = 98)	*P* value
Age (y)	54.8 ± 10.8	56.2 ± 11.3	0.426
Sex			0.273
Male	36 (62.1)	52 (53.1)	
Female	22 (37.9)	46 (46.9)	
Ulcer area (cm^2^)			0.856
≤ 5	8 (13.8)	16 (16.3)	
5 ~ 10	32 (55.2)	55 (56.1)	
> 10	18 (31.0)	27 (27.6)	
Ulcer duration (week)			0.043
≤ 6	10 (17.2)	11 (11.2)	
6 ~ 10	33 (56.9)	42 (42.9)	
> 10	15 (25.9)	45 (45.9)	
Wagner grade			0.005
II	14 (24.1)	6 (6.1)	
III	35 (60.3)	73 (74.5)	
IV	9 (15.6)	19 (19.4)	
Severity of infection			0.003
Mild	14 (24.1)	6 (6.1)	
Moderate	28 (48.3)	66 (67.3)	
Severe	16 (27.6)	26 (26.6)	
Recurrent DFU	12 (20.7)	39 (39.8)	0.014
Detection rate of drug resistant bacteria	13 (22.4)	38 (38.8)	0.035
Outcome of ulcer after 8 weeks			0.031
Healing	37 (63.8)	45 (45.9)	
Non-healing	21 (36.2)	53 (54.1)	
Amputation rate (%)			0.035
Amputation	9 (15.5)	30 (30.6)	
Non-amputation	49 (84.5)	68 (69.4)	

Data are presented mean ± standard deviations or numbers (%); Differences between two groups analyzed using t test or *x*^*2*^ test. The cut-off point of serum vitamin D level for grouping was 10.3 ng/mL

*DFU* diabetic foot ulcer, *25(OH)VD* 25-hydroxyvitamin D

**Table 4:** Relationship between the levels of T-VDR expression and the clinical features of DFU patients [( ± s), n (%)]

	High expression group (n = 53)	Low expression group (n = 103)	*P* value
Age (y)	55.1 ± 10.8	55.8 ± 11.3	0.613
Sex			0.162
Male	34 (64.2)	54 (52.4)	
Female	19 (35.8)	49 (47.6)	
Ulcer area (cm^2^)			0.787
≤ 5	7 (13.2)	17 (16.5)	
5 ~ 10	30 (56.6)	57 (55.4)	
> 10	16 (30.2)	29 (28.1)	
Ulcer duration (week)			0.014
≤ 6	9 (17.0)	12 (11.7)	
6 ~ 10	32 (60.4)	43 (41.7)	
> 10	12 (22.6)	48 (46.6)	
Wagner grade			0.030
II	12 (22.6)	8 (7.7)	
III	32 (60.3)	76 (73.9)	
IV	9 (7.1)	19 (18.4)	
Severity of infection			0.021
Mild	12 (22.6)	8 (7.8)	
Moderate	26 (49.1)	68 (66.0)	
Severe	15 (28.3)	27 (26.2)	
Recurrent DFU	11 (20.8)	40 (38.8)	0.023
Detection rate of drug resistant bacteria	10 (18.9)	41 (39.8)	0.012
Outcome of ulcer after 8 weeks			0.016
Healing	35 (66.0)	47 (45.6)	
Non-healing	18 (34.0)	56 (54.4)	
Amputation rate (%)			0.040
Amputation	8 (15.1)	31 (30.1)	
Non-amputation	45 (84.9)	72 (69.9)	

Data are presented mean ± standard deviations or numbers (%); Differences between two groups analyzed using t test or *x*^*2*^ test. The cut-off point of vitamin D receptor expression level for grouping was 2.68

*DFU* diabetic foot ulcer

**Table 5:** Relationship between the levels of T-VDR expression and the clinical features of DFO patients [( ± s), n (%)]

	High expression group (n = 32)	Low expression group (n = 52)	*P* value
Age (y)	55.6 ± 10.5	56.4 ± 11.1	0.482
Sex			0.830
Male	18 (56.3)	28 (53.8)	
Female	14 (43.7)	24 (46.2)	
Ulcer area (cm^2^)			0.786
≤ 5	3 (9.4)	7 (13.5)	
5 ~ 10	18 (56.3)	30 (57.7)	
> 10	11 (34.3)	15 (28.8)	
Ulcer duration (week)			0.037
≤ 6	4 (12.5)	5 (9.6)	
6 ~ 10	18 (56.3)	16 (30.8)	
> 10	10 (31.2)	31 (59.6)	
Wagner grade			0.011
II	0 (0.0)	0 (0.0)	
III	25 (75.0)	25 (46.1)	
IV	7 (18.7)	27 (48.1)	
Severity of infection			0.041
Mild	0 (0.0)	0 (0.0)	
Moderate	24 (75.0)	27 (51.9)	
Severe	8 (25.0)	25 (48.1)	
Recurrent DFU	8 (25.0)	26 (50.0)	0.023
Detection rate of drug resistant bacteria	7 (21.9)	27 (51.9)	0.006
Outcome of ulcer after 8 weeks			0.021
Healing	20 (62.5)	19 (36.5)	
Non-healing	12 (37.5)	33 (63.5)	
Amputation rate (%)			0.039
Amputation	5 (15.6)	19 (36.5)	
Non-amputation	27 (84.4)	33 (63.5)	

Data are presented mean ± standard deviations or numbers (%); Differences between two groups analyzed using t test or *x*^*2*^ test. The cut-off point of vitamin D receptor expression level for grouping was 2.68

*DFU* diabetic foot ulcer, *DFO* diabetic foot osteomyelitis

### Correlation between serum 25(OH)VD levels and other clinical parameters

**Fig. 2:**
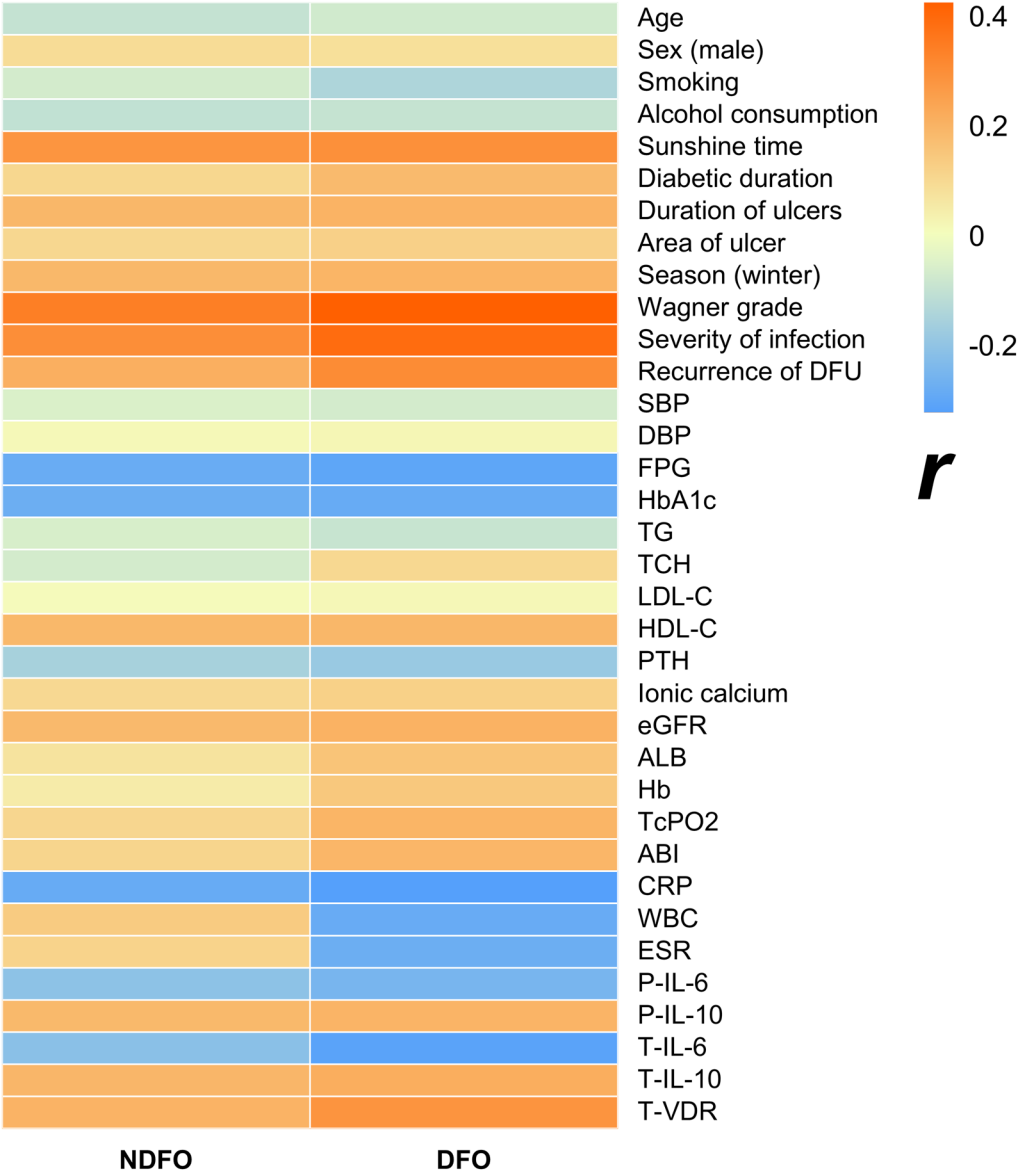
The correlations between serum 25(OH)VD levels and other clinical parameters in NDFO and DFO group (*r*). Pearson correlation analysis showed that serum 25(OH)VD levels were positively correlated with mean sunshine duration per day, ulcer time, Wagner grade, infection severity, recurrence of foot ulcer, HDL-C, P-IL-10, T-IL-10, and T-VDR levels in NDFO group and DFO group, (*P* < 0.05), but negatively correlation with season (winter), FPG, HbA1c, CRP, P-IL-6, and T-IL-6 levels (*P* < 0.05). Serum 25(OH)VD levels were also positively correlated with TcPO2 and ABI in the DFO group (*P* < 0.05), and negatively correlated with WBC, ESR (*P* < 0.05). *NDFO* diabetic foot ulcer without osteomyelitis, *DFO* diabetic foot osteomyelitis, *DFU* diabetic foot ulcer, *SBP* systolic blood pressure, *DBP* diastolic blood pressure, *FPG* fasting plasma glucose, *HbA1c* glycated hemoglobin A1c, *TG* triacylglycerol, *TCH* total cholesterol, *LDL-C*low-density lipoprotein cholesterol, *HDL-C*high-density lipoprotein cholesterol, *PTH* parathyroid hormone, *eGFR* estimated glomerular filtration rate, *ALB* serum albumin, *Hb* haemoglobin, *TcPO2* transcutaneous oxygen pressure, *ABI* ankle brachial index, *CRP* C-reactive protein, *WBC* white blood cell, *ESR* erythrocyte sedimentation rate, *IL* interleukin, *T-IL-6* IL-6 mRNA expression in wound margin tissue, *T-IL-10* IL-10 mRNA expression in wound margin tissue, *T-VDR*:Vitamin D receptor mRNA expression in wound margin tissue, *IL* interleukin, *P-IL-6* IL-6 level in peripheral blood, *P-IL-10* IL-10 level in peripheral blood.*T-IL-6* IL-6 mRNA expression in wound margin tissue, *T-IL-10* IL-10 mRNA expression in wound margin tissue,*T-VDR* vitamin D receptor mRNA expression in wound margin tissue

In NC group and T2DM group, serum 25(OH)VD levels were positively correlated with mean sunshine duration per day (*P* < 0.05). Additionally, in the T2DM group, serum 25(OH)VD levels were positively correlated with HDL-C and eGFR levels (*P* < 0.05), and negatively correlated with FPG, HbA1c levels, and season (winter) (*P* < 0.05). There was no significant correlation between serum 25(OH)VD levels and other clinical parameters in T2DM group and NC group (*P* > 0.05) (Additional file 1: Fig. S1). As for the NDFO group and the DFO group, serum 25(OH)VD levels were positively correlated with mean sunshine duration per day, ulcer time, Wagner grade, infection severity, recurrence of foot ulcer, HDL-C, P-IL-10, T-IL-10, and T-VDR expression levels (*P* < 0.05). However, serum 25(OH)VD levels showed a negative correlation with season (winter), FPG, HbA1c, CRP, P-IL-6, and T-IL-6 expression levels (*P* < 0.05). In addition, in the DFO group, serum 25(OH)VD levels were also positively correlated with TcPO2 and ABI (*P* < 0.05), and negatively correlated with WBC, ESR (*P* < 0.05). No significant correlation between serum 25(OH)VD levels and other clinical parameters was observed in the NDFO group and the DFO group (*P* > 0.05) (Fig. 2).

### Risk factor analysis for diabetes foot ulcer and diabetes foot osteomyelitis

In diabetic patients, multivariate unconditional logistic regression was performed with DFU as the dependent variable and sex, age, duration of diabetes, regular smoking, long-term alcohol consumption, mean sunshine duration per day in the recent 3 months, SBP, DBP, FPG, HbA1c, TG, TCH, LDL-C, HDL-C, eGFR, ALB, Hb, TcPO2, ABI, CRP, WBC, ESR, serum 25(OH)VD level, P-IL-6 level and P-IL-10 level as independent variables, respectively. The results showed that the duration of diabetes, HbA1c, eGFR, TcPO2, CRP, and low serum 25(OH)VD level was independent risk factors for DFU (Table 6). Also, in the DFU patients, multivariate unconditional logistic regression was performed with DFO as the dependent variable and sex, age, duration of diabetes, duration of foot ulcer, area of foot ulcer, regular smoking, long-term drinking, mean sunshine duration per day in the recent 3 months, Wagner grade, the severity of wound infection, detection of drug-resistant bacteria, SBP, DBP, FPG, HbA1c, TG, TCH, LDL-C, HDL-C, eGFR, ALB, Hb, TcPO2, ABI, CRP, WBC, ESR, serum 25(OH)VD level, P-IL-6 level, P-IL-10 level, T-IL-6, T-IL-10, and T-VDR expression levels as independent variables. The final analysis indicated that independent risk factors for DFO included the duration of foot ulcer, the severity of infection, HbA1c, ABI, WBC, ESR, low serum 25(OH)VD level, and low level of T-VDR expression (Table 7).

**Table 6:** The multivariate logistic regression analysis of risk factors of diabetic foot ulcer

Variable	β	SE	Wald	OR	95% CI	*P* value
Duration of diabetes (y)	0.47	0.29	9.56	4.13	1.64 ~ 12.15	< 0.001
HbA1c (%)	0.34	0.17	3.51	1.26	1.13 ~ 6.77	0.031
eGFR (ml/min/1.73m^2^)	0.26	0.14	2.97	1.13	1.04 ~ 6.23	0.046
TcPO2 (mmHg)	0.41	0.27	4.13	1.69	1.18 ~ 9.84	0.019
CRP (mg/L)	0.45	0.23	3.14	1.22	1.09 ~ 7.96	0.025
25 (OH) VD (ng/ml)	0.62	0.34	5.43	2.42	1.37 ~ 10.88	0.008

Multivariate unconditional logistic regression analysis adjusted for sex, age, duration of diabetes, frequent smoking, long term drinking, mean sunshine time per day, SBP, DBP, FPG, HbA1c, TG, TCH, LDL-C, HDL-C, eGFR, ALB, Hb, TcPO2, ABI, CRP, WBC, ESR, P-IL-6, P-IL-10, serum 25 (OH) VD

*SBP* systolic blood pressure, *DBP* diastolic blood pressure, *FPG* fasting plasma glucose, *HbA1c* glycated hemoglobin A1c, *TG* triacylglycerol, *TCH* total cholesterol, *LDL-C* low-density lipoprotein cholesterol, *HDL-C* high-density lipoprotein cholesterol, *eGFR* Estimated glomerular filtration rate, *ALB* serum albumin, *Hb* hemoglobin, *TcPO2* transcutaneous oxygen pressure, *ABI* ankle brachial index, *CRP* C-reactive protein, *WBC* white blood cell, *ESR* erythrocyte sedimentation rate, *P-IL-6* IL-6 level in peripheral blood, *P-IL-10* IL-10 level in peripheral blood, *25(OH)VD* 25-hydroxyvitamin D

**Table 7:** The multivariate logistic regression analysis of risk factors of diabetic foot osteomyelitis

Variable	β	SE	Wald	OR	95% CI	*P* value
Duration of ulcers (w)	0.69	0.35	9.22	4.81	1.21 ~ 11.63	< 0.001
Severity of infection	0.52	0.29	5.83	2.07	1.09 ~ 10.34	0.012
HbA1c (%)	0.41	0.23	6.03	2.35	1.24 ~ 9.26	0.009
ABI	0.38	0.19	3.59	1.46	1.12 ~ 7.49	0.027
WBC (× 10^9^)	0.21	0.11	2.87	1.19	1.04 ~ 6.18	0.041
ESR (mm/h)	0.46	0.25	4.96	1.87	1.13 ~ 9.15	0.018
25 (OH) VD (ng/ml)	0.58	0.26	7.15	3.05	1.07 ~ 12.53	0.001
T-VDR	0.49	0.17	5.12	2.83	1.15 ~ 9.97	0.004

Multivariate unconditional logistic regression analysis adjusted for for sex, age, duration of diabetes, duration of ulcers, frequent smoking, long term drinking, mean sunshine time per day, Wagner grade, severity of infection, detection rate of drug resistant bacteria, SBP, DBP, FPG, HbA1c, TG, TCH, LDL-C, HDL-C, eGFR, ALB, Hb, TcPO2, ABI, CRP, WBC, ESR, P-IL-6, P-IL-10, serum 25 (OH)VD

*SBP* systolic blood pressure, *DBP* diastolic blood pressure, *FPG* fasting plasma glucose, *HbA1c* glycated hemoglobin A1c, *TG* triacylglycerol, *TCH* total cholesterol, *LDL-C* low-density lipoprotein cholesterol, *HDL-C* high-density lipoprotein cholesterol, *eGFR* Estimated glomerular filtration rate, *ALB* serum albumin, *Hb* hemoglobin, *TcPO2* transcutaneous oxygen pressure, *ABI* ankle brachial index, *CRP* C-reactive protein, *WBC* white blood cell, *ESR* erythrocyte sedimentation rate, *P-IL-6* IL-6 level in peripheral blood, *P-IL-10* IL-10 level in peripheral blood, *25(OH)VD* 25-hydroxyvitamin D, *T-VDR* vitamin D receptor mRNA expression in wound margin tissue

### Mark verification

**Fig. 3:**
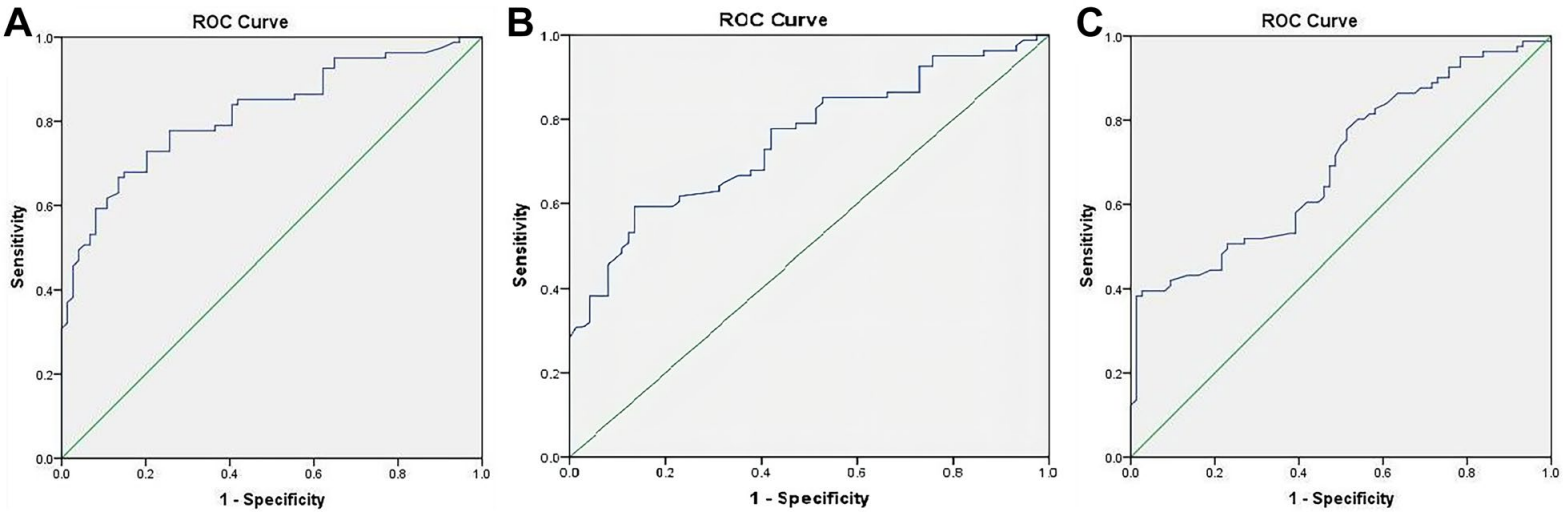
The biomarker potential of serum 25(OH)VD for DFU and DFO and that of T-VDR for DFO. **A** Serum 25(OH)VD distinguished DFU patients from controls with area under curve (AUC) of 0.821 (95% CI: 0.754–0.886, *P* < 0.001). **B** Serum 25(OH)VD distinguished DFO patients from DFU patients (AUC: 0.786; 95%CI: 0.643–0.867, *P* < 0.001). **C** T-VDR distinguished DFO patients from DFU patients (AUC: 0.703; 95%CI: 0.618–0.853, *P* < 0.001). *25(OH)VD* 25-hydroxyvitamin D, *DFU* diabetic foot ulcer, *DFO* diabetic foot osteomyelitis, *T-VDR* Vitamin D receptor mRNA expression in wound margin tissue. [Color figure can be viewed at wileyonlinelibrary.com]

To further investigate the potential value of 25(OH)VD in the diagnosis of DFU, the levels of serum 25(OH)VD were evaluated in 256 independent peripheral blood samples (including 100 samples of patients with T2DM and 156 with DFU). The sensitivity and specificity of serum 25(OH)VD in the diagnosis of DFU were evaluated using the ROC curve. The results showed that the area under the ROC curve (AUC) of serum 25(OH)VD in the diagnosis of DFU was 0.821 (95% confidence interval [CI], 0.754–0.886, *P* < 0.001), the optimal cutoff point of serum 25(OH)VD was 12.5 ng/mL, the sensitivity was 96.35%, and the specificity was 95.82% (Fig. 3A). In order to further explore the potential value of serum 25(OH)VD and T-VDR to establish the diagnosis of DFO, the levels of serum 25(OH)VD and T-VDR expression were evaluated in 156 cases peripheral blood samples and wound margin tissue samples in independent groups; these samples included samples of 72 patients with DFU without osteomyelitis and of 84 patients with DFO. The sensitivity and specificity of levels of serum 25(OH)VD and T-VDR expression in the diagnosis of DFO were evaluated using the ROC curve. The results showed that the AUC of serum 25(OH)VD level in the diagnosis of DFO was 0.786 (95%CI, 0.643–0.867, *P* < 0.001), the optimal cut-off point of serum 25(OH)VD level was 8.6 ng/mL, the sensitivity was 94.61%, and the specificity was 96.13% (Fig. 3B). The AUC of T-VDR expression level in diagnosing DFO was 0.703 (95%CI, 0.618–0.853, *P* < 0.001), the optimal cut-off point of T-VDR expression level was 1.14, the sensitivity was 94.23%, and the specificity was 95.82% (Fig. 3C).

## Discussion

In the present study, T2DM patients showed lower levels of serum 25(OH)VD and higher proportions of vitamin D deficiency and insufficiency than individuals with normal glucose tolerance. In addition, the levels of serum 25(OH)VD in DFU patients were also significantly lower than that of diabetes patients without DFU. But it has to be said on condition that the foot ulcer infection progresses to diabetic foot osteomyelitis, the levels of serum 25(OH)VD and T-VDR expression will decrease sharply, and the proportion of vitamin D deficiency will also increase significantly. Multivariate logistic regression analysis revealed that decreased serum 25(OH)VD level was an independent risk factor for DFU and DFO, and decreased T-VDR expression level was an independent risk factor for DFO. Further analysis showed that the levels of serum 25(OH)VD and T-VDR expression of patients with T2DM could be used as markers to predict DFU and DFO, and were closely related to the healing rate of foot ulcer and amputation rate. DFU with low serum 25(OH)VD level and low T-VDR expression level had a lower healing rate and higher risk of amputation, indicating that vitamin D deficiency and decreased VDR expression level are not only strong risk factors for the onset of DFU and DFO but also as potential biomarkers for the prognosis of DFU and DFO. To the best of our knowledge, this is the first systematic study on the relationship between changes in serum 25(OH)VD level and T-VDR expression level with the onset and treatment outcomes of DFU and DFO in patients with T2DM.

A prospective cohort study from Australia reported that 55.7% of hospitalized patients with DFU presented with vitamin D deficiency [18]. According to a retrospective study in the United States, 78% of patients with DFU undergoing foot and ankle surgery showed vitamin D deficiency or insufficiency [19]. In addition, a single-center retrospective study of inpatient DFU patients from China showed vitamin D deficiency in 86.8% of patients with DFU [20]. The results of the present study demonstrated that vitamin D deficiency was 71.8% in DFU and 82.1% in DFO. Additionally, vitamin D deficiency and insufficiency became more pronounced as DFU progressed, with a Wagner grade of 4 and the presence of DFU with severe infection reaching 100%. Therefore, through these results, it is evident that vitamin D deficiency and insufficiency are prevalent in DFU and may be associated with the severity of DFU. Some studies reported that the increase of Wagner grade resulted in a reduction in the levels of 25(OH)VD in the peripheral blood of patients with DFU [20], which supported our findings.

In the present study, we noted that the levels of serum 25(OH)VD were significantly lower in patients with T2DM than in individuals in the NC group. Correlation analysis showed that serum 25(OH)VD levels were negatively correlated with FPG and HbA1c, and positively correlated with HDL-C, both in T2DM alone and in T2DM with foot ulcers, which suggested that the levels of vitamin D in patients with T2DM were intrinsically correlated with glucose and lipid metabolism, which were consistent with previous findings [21, 22]. Furthermore, the outcomes of the present study showed that the levels of serum 25(OH)VD were negatively correlated with the duration of foot ulcers, Wagner grade of foot ulcers, the severity of wound infection (including IDSA grade, inflammatory indicators including CRP, WBC, ESR, and IL-6 levels, ect.), the recurrence rate of foot ulcers, and detection rate of drug-resistant bacteria in wounds in patients with DFU. However, the levels of serum 25(OH)VD were positively correlated with the levels of anti-inflammatory factor IL-10. Previous studies have confirmed that drug-resistant bacteria are risk factors for the recurrence of DFU [23, 24]. Therefore, the results of the current study suggested that the levels of serum 25(OH)VD in patients with DFU is related to the severity of DFU, the severity and complexity of wound infection, and the risk of foot ulcer recurrence. A study has found [25] that vitamin D can promote the differentiation of dendritic cells and regulatory T cells by binding to VDR, and reduce the Th17 response of T helper cells and the secretion of inflammatory cytokines, in order to play an anti-inflammatory role. In addition, vitamin D can enhance the expression of antimicrobial peptides in a variety of cells, which can help eliminate microorganisms, inhibit proinflammatory responses, and enhance anti-inflammatory responses [10]. These observations are consistent with the conclusions presented in the current study. Moreover, in the NC, T2DM, and DFU groups of the present study, we observed that the levels of serum 25(OH)VD were associated with the mean sunshine duration and season, and the serum 25(OH)VD levels were relatively lower in the subjects with short sunshine time and winter. In most areas of China with relatively less sunshine in winter and spring, the average levels of serum 25(OH)VD were reportedly low [26]. A study from Germany suggested that sunshine duration was closely related to the levels of serum 25(OH)VD; additionally, a shorter sunshine duration was associated with lower serum 25(OH)VD levels [27]. Specifically, in this study, the sunshine duration in the DFU group was lower than that in the NC group and T2DM group, which may be correlated to the inconvenience of activity and the reduction of outdoor exercise attributable to DFU.

Notably, in this study, the duration of foot ulcer in the DFU group was at least 4 weeks, which indicated the presence of chronic refractory wound. The clinical features include a long duration of diabetes and poor long-term blood glucose control in combination with different degrees of chronic kidney disease, abnormal lipid metabolism, peripheral vascular disease, and infectious inflammation. Wagner classification was predominantly grades 3–4. Multivariate regression analysis showed that duration of diabetes, HbA1c, TcPO2, CRP, and eGFR were independent factors influencing the occurrence of foot ulcers, which were consistent with previous findings [28, 29]. Further analysis confirmed that compared with the T2DM group, serum 25(OH)VD levels in the DFU group were significantly lower, and the proportion of vitamin D deficiency was higher; the serum 25(OH)VD levels were negatively correlated with the duration of ulcer, and positively correlated with the ulcer healing rate after 8 weeks of treatment as well amputation rate. Multivariate regression analysis showed that low levels of serum 25(OH)VD were an independent risk factor for foot ulcers. It is suggested that 25(OH)VD may be involved in the occurrence of diabetic foot ulcers and can be used as a marker for the prognosis of DFU. Multiple studies have reported that vitamin D deficiency is significantly associated with a high prevalence of DFU [30, 31]. A meta-analysis reported that vitamin D levels were significantly reduced in patients with DFU, and severe vitamin D deficiency was significantly associated with an increased risk of DFU [6]. The basic elements of diabetic foot are diabetic lower extremity arterial disease and peripheral neuropathy. Low levels of serum 25(OH)VD in patients with T2DM are closely related to the occurrence of arterial lesions in the lower extremity [32]. Investigation has shown that the incidence of vitamin D deficiency in patients with T2DM with peripheral neuropathy in China is 80%, and vitamin D deficiency is an independent risk factor for diabetic peripheral neuropathy [33]. These results support our findings.

At present, it is generally believed that the adverse effects of the high glucose environment in the body, the obvious changes in the skin microenvironment, the low immune function, the reduced antibacterial ability of the wound, the damaged function of keratinocytes and fibroblasts, and the insufficient angiogenesis of the wound are all important influencing factors for the difficulty of DFU healing [34]. However, the physiological relationship between the levels of vitamin D and VDR expression and wound healing in diabetes have not been fully understood. Gonzalez-Curiel et al. reported that 1,25(OH)2D3 can upregulate the expression of DEFB4 and CAMP genes in primary keratinocytes from DFU, increase the production of HBD-2 and LL-37 in cell-culture supernatant, and improve the migration ability and antibacterial activity of keratinocytes, thus promoting wound healing[35]. Xiong et al. found that 1,25(OH)2D3 can inhibit excessive autophagy and oxidative stress in vascular endothelial cells caused by advanced glycation end products (AGEs) through the PI3K/Akt pathway, thereby promoting angiogenesis in a high-glucose environment [36], which is conducive to wound healing. Animal experiments have demonstrated that vitamin D can improve wound healing in diabetic mice by inhibiting the expression of inflammatory genes such as IL-6 and IL-1β mediated by NF-κB [37] and inhibiting endoplasmic reticulum stress [38]. In vitro studies have confirmed that 1,25(OH)2D3 can promote the transformation of M1 macrophages to M2 macrophages induced by high glucose through the VDR-PPARγ signaling pathway, and enhance the bactericidal activity of macrophages against *Pseudomonas aeruginosa* [39]. Recent studies have confirmed that 1,25(OH)2D3 can upregulate the complement receptor immunoglobulin (CRIg) in macrophages—which play an important role in innate immunity—and enhance the phagocytosis of macrophages against Staphylococcus aureus and Candida albicans [40]. In the wound infection model, topical application of 1,25(OH)2D3 to the skin of CAMP transgenic mice can induce CAMP expression and increase the killing effect of Staphylococcus aureus [41]. Clinical studies have shown a significant association between the functional variation of the VDR gene FokI and DFU and oxidative stress. Patients with T2DM carrying the T allele of FokI polymorphism have increased levels of oxidative stress and a higher risk of foot ulcers [42]. Although a series of studies have explored the possible mechanism of vitamin D and VDR involvement in the occurrence and healing of diabetic wounds from different aspects, additional studies are warranted to further clarify in the future.

Further analysis found that compared with simple foot ulcers, patients with foot ulcers combined with osteomyelitis had a longer duration of diabetes and foot ulcers, higher Wagner grade of foot ulcers, poorer glycaemic control status, as well as worse limb blood supply status, more severe degree of infection, more severe infection along with higher proportion of drug-resistant infection. Multivariate regression analysis showed that the duration of foot ulcer, the severity of wound infection, HbA1c, ABI, WBC, and ESR were independent influencing factors for the occurrence of foot ulcer complicated with osteomyelitis, which was consistent with previous findings [28, 43]. More details, further analysis showed that the serum 25(OH)VD levels and T-VDR expression levels in the DFO group were further decreased compared with the NDFO group; the serum 25(OH)VD levels in patients with DFO were positively correlated with T-VDR expression levels; the levels of serum 25(OH)VD and T-VDR expression were negatively correlated with the amputation rate of foot ulcers with osteomyelitis. Multivariate regression analysis showed that low level of serum 25(OH)VD and VDR expression were independent risk factors for foot ulcer with osteomyelitis. Meanwhile, it suggested that 25(OH)VD and VDR might be involved in the occurrence of DFO and can be used as a marker for the risk of DFO amputation. A cross-sectional study from India showed that vitamin D deficiency was associated with poor prognosis in co-infected DFU, leading to osteomyelitis, amputation, and increased risk of death [9], thereby supporting our findings. DFO is mostly chronic osteomyelitis, which is caused by bone destruction and dead bone formation caused by infection. Therefore, the ability of infection clearance, osteoblast-mediated bone formation, and osteoclast-regulated bone resorption are crucial to the repair of bone injury, and can directly affect the clinical outcome of osteomyelitis. Shekhar C reported that the local application of vitamin D3 particles in open infectious wounds could increase the expression of antimicrobial peptides (cathelicidin) in wounds, enhance the antibacterial ability of wounds, promote bone growth, and restore bone mineral density [44]. A retrospective cohort study of patients with bone and joint infection showed that low level of serum 25(OH)VD could reduce the success rate of treatment for bone infection [45]. Li et al. found that 1,25(OH)2D3 could increase the expression of the anti-inflammatory factor IL-10 by reducing the expression of genes related to osteoclasts (c-Fos, NFATc1, CTSK, and TRAP) and pro-inflammatory factors (IL-6, IL-12p40, and TNF-α), thereby reducing bone resorption in a mouse skull model infected with *Porphyromonas gingivalis* [46]. Studies have revealed that the binding of 1,25(OH)2D3 to VDR can inhibit the nuclear transmigration of NF-KB-P65, IL-8, IL-6, TNFα, and NR4A2 transcripts in human bone marrows derived mesenchymal stem cells, demonstrating the anti-MRSA infection effect of active vitamin D [47]. Jiang et al. demonstrated that both TaqI (rs731236) and FokI (rs2228570) polymorphisms of the VDR gene can increase the risk of chronic osteomyelitis in the Chinese population [48]. Although some studies have confirmed that vitamin D and VDR play a role in the pathogenesis of osteomyelitis, further studies are warranted to elucidate the mechanism of 25(OH)VD and VDR involved in the occurrence of DFO.

It turned out that the levels of 25(OH)VD in peripheral blood could be used as a biomarker for predicting and diagnosing mild cognitive impairment [49] and active Crohn's disease [50]. In addition, VDR expression can be used as a biomarker to predict sepsis mortality [51] and bone metastasis of breast cancer [52]. In the present study, we noted that serum 25(OH)VD level in patients with T2DM can be used as a potential biomarker for predicting DFU and DFO. In addition, the T-VDR expression was also found to be a potential biomarker for predicting DFO. What is more, we also discovered that the levels of serum 25(OH)VD and T-VDR expression were negatively correlated with the duration of DFU, the severity of foot ulcer, the recurrence rate of foot ulcer, the detection rate of drug-resistant bacteria in the wound and the amputation rate of simple foot ulcers or foot ulcers combined with osteomyelitis. On the contrary, it also represents positively correlated with the healing rate of simple foot ulcers or foot ulcers combined with osteomyelitis after 8 weeks of treatment. Therefore, the forementioned results illustrate the functionality of serum 25(OH)VD level and T-VDR expression for the diagnosis and prognosis of DFU and DFO. Nonetheless, further studies are needed to identify the reasons for the decreased levels of serum 25(OH)VD and T-VDR expression with DFU and DFO.

In conclusion, this study found that the decreased levels of serum 25(OH)VD and T-VDR expression of type 2 diabetes patients was closely associated with the occurrence, development and prognosis of DFU. Yet when it comes to the shortcomings of this study, the main shortcoming as follows: (1) it is a single-center study with a relatively small sample size, and selection bias. (2) There are differences in the duration of diabetes and blood glucose levels between the T2DM group and the DFU group, which may affect the expression profile of cytokines, and may affect the results of multivariate unconditional logistic regression as a confounding factor. Therefore, the results of this study need to be further confirmed. (3) The influence of lifestyle, diet, and other factors on serum 25(OH)VD level was not considered. (4) The causal relationship between 25(OH)VD, VDR, and the pathogenesis of DFU (including DFO) could not be clarified in the present study. In the future, more studies are needed to further explore the role of 25(OH)VD and VDR in DFU (including DFO) and to evaluate whether 25(OH)VD and VDR can become new therapeutic targets for DFU (including DFO).

## Supplementary Information

**Additional file 1: Figure S1.** The correlations between serum 25(OH)VD levels and other clinical parameters in the NC and T2DM group (*r*). Pearson correlation analysis showed that serum 25(OH)VD levels were positively correlated with mean sunshine duration per day in NC group (*P* < 0.05) and were positively correlated with mean sunshine duration per day, HDL-C and eGFR levels in T2DM group (*P* < 0.05),but negatively correlated with FPG, HbA1c levels, and season (winter) (*P* < 0.05). NC: normal control group; T2DM: type 2 diabetes group group;SBP: systolic blood pressure; DBP: diastolic blood pressure; FPG: fasting plasma glucose; HbA1c: glycated hemoglobin A1c; TG:triacylglycerol; TCH:total cholesterol; LDL-C:low-density lipoprotein cholesterol; HDL-C:high-density lipoprotein cholesterol; PTH: parathyroid hormone; eGFR: estimated glomerular filtration rate; ALB: serum albumin; Hb: haemoglobin; TcPO2: transcutaneous oxygen pressure; ABI: ankle brachial index; CRP: C-reactive protein; WBC: white blood cell; ESR: erythrocyte sedimentation rate; IL: interleukin; P-IL-6: IL-6 level in peripheral blood; P-IL-10: IL-10 level in peripheral blood.

**Additional file 2: Table S1.** Detection method of observation indices.

**Additional file 3: Table S2.** Specific primers and reaction conditions in qRT-PCR.

**Additional file 4: Table S3.** Comparison of vitamin D nutritional status in diabetes foot ulcers with different Wagner grades and infection severity.

## Data Availability

All data generated or analysed during this study and supporting our fndings are included and can be found in the manuscript. The raw data can be provided by corresponding author on reasonable request.

## Abbreviations

T2DM: Type 2 diabetes mellitus
DFU: Diabetic foot ulcer
DFO: Diabetic foot osteomyelitis
VDR: Vitamin D receptor
25(OH)VD: 25-Hydroxyvitamin D
ABI: Ankle-brachial index
OGTT: Oral glucose tolerance test
FPG: Fasting plasma glucose
NPWT: Negative pressure wound treatment
MDT: Multidisciplinary team
SBP: Systolic blood pressure
DBP: Diastolic blood pressure
IDSA: Infectious Disease Society of America
TcPO_2_: Transcutaneous oxygen pressure
HbA1c: Glycosylated hemoglobin A1c
WBC: White blood cell
CRP: C-reactive protein
ESR: Erythrocyte sedimentation rate
PTH: Parathyroid hormone
TCH: Total cholesterol
TG: Triglyceride
HDL-C: High-density lipoprotein cholesterol
LDL-C: Low-density lipoprotein cholesterol
ALB: Serum albumin
Hb: Hemoglobin
IL-6: Interleukin-6
IL-10: Interleukin-10
eGFR: Estimated glomerular filtration rate
qRT-PCR: Quantitative real-time polymerase chain reaction
T-VDR: Vitamin D receptor mRNA in wound margin tissues
T-IL-6: Interleukin-6 mRNA in wound margin tissues
T-IL-10: Interleukin-10 mRNA in wound margin tissues
ROC: Receiver operating characteristic
P-IL-10: Interleukin-10 levels in peripheral blood
P-IL-6: Interleukin-6 levels in peripheral blood
AGEs: Advanced glycation end products
CRIg: Complement receptor immunoglobulin
