# Supplementary figures and images for: Level of 25-hydroxyvitamin D and vitamin D receptor in diabetic foot ulcer and factor associated with diabetic foot ulcers
Source: Diabetol Metab Syndr. 2023 Feb 24;15:30. doi: 10.1186/s13098-023-01002-3 (PMC9951493; doi:10.1186/s13098-023-01002-3)

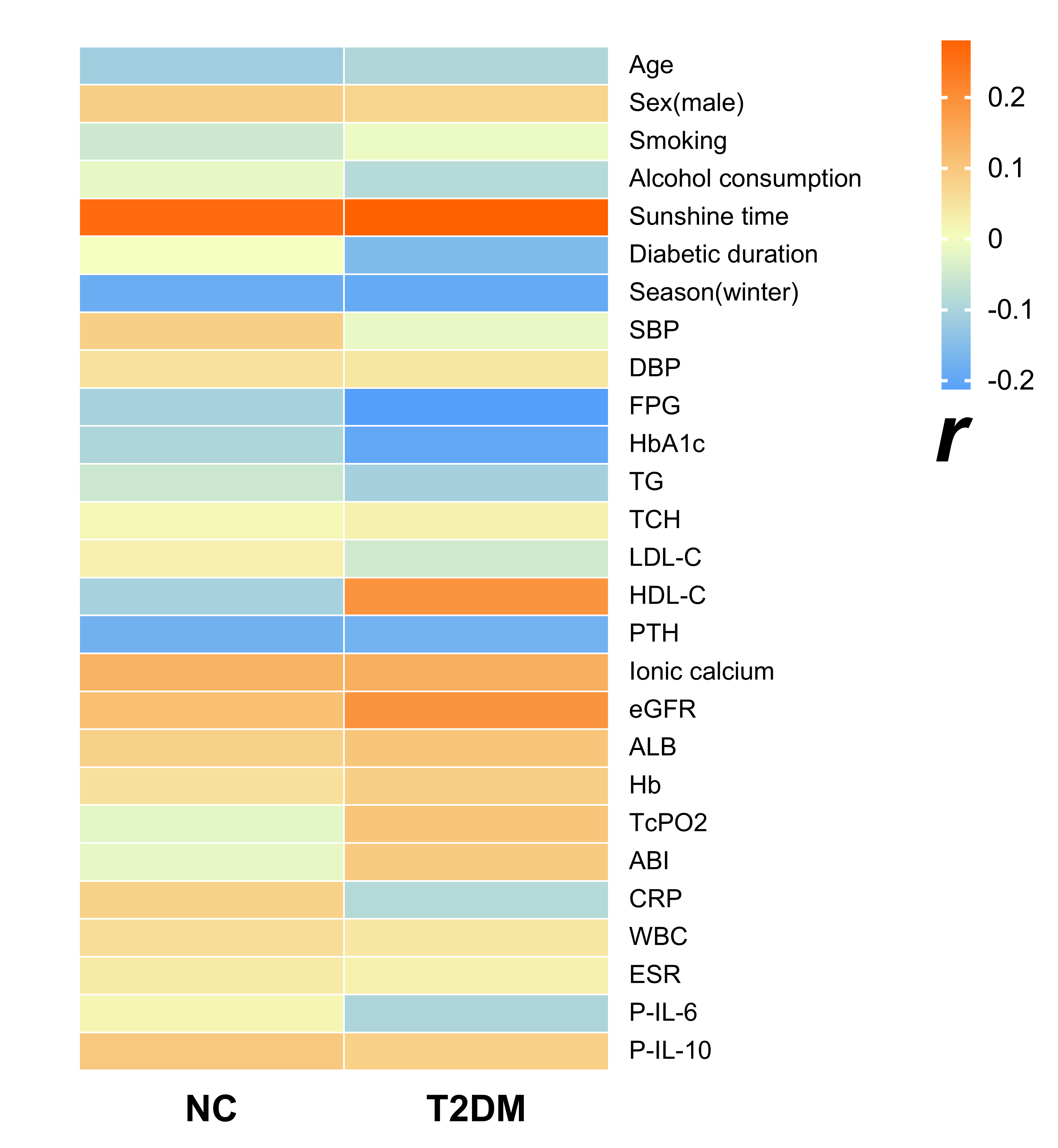

Supplement: Supplementary file 1 — Additional file 1: Figure S1. The correlations between serum 25(OH)VD levels and other clinical parameters in the NC and T2DM group (r). Pearson correlation analysis showed that serum 25(OH)VD levels were positively correlated with mean sunshine duration per day in NC group (P < 0.05) and were positively correlated with mean sunshine duration per day, HDL-C and eGFR levels in T2DM group (P < 0.05),but negatively correlated with FPG, HbA1c levels, and season (winter) (P < 0.05). NC: normal control group; T2DM: type 2 diabetes group group;SBP: systolic blood pressure; DBP: diastolic blood pressure; FPG: fasting plasma glucose; HbA1c: glycated hemoglobin A1c; TG:triacylglycerol; TCH:total cholesterol; LDL-C:low-density lipoprotein cholesterol; HDL-C:high-density lipoprotein cholesterol; PTH: parathyroid hormone; eGFR: estimated glomerular filtration rate; ALB: serum albumin; Hb: haemoglobin; TcPO2: transcutaneous oxygen pressure; ABI: ankle brachial index; CRP: C-reactive protein; WBC: white blood cell; ESR: erythrocyte sedimentation rate; IL: interleukin; P-IL-6: IL-6 level in peripheral blood; P-IL-10: IL-10 level in peripheral blood. [file 13098_2023_1002_MOESM1_ESM.tif]
