# Supplementary material for: Level of 25-hydroxyvitamin D and vitamin D receptor in diabetic foot ulcer and factor associated with diabetic foot ulcers
Source: Diabetol Metab Syndr. 2023 Feb 24;15:30. doi: 10.1186/s13098-023-01002-3 (PMC9951493; doi:10.1186/s13098-023-01002-3)
Supplement: Supplementary file 2 — Additional file 2: Table S1. Detection method of observation indices. [file 13098_2023_1002_MOESM2_ESM.doc]

**Table S1** Detection method of observation indices

| Observation indices | Detection method |
| --- | --- |
| FPG | glucose oxidase method |
| TCH, TG, HDL-C, and LDL-C | oxidase-linked colorimetry |
| HbA1c | high-pressure liquid chromatography |
| CRP | latex-enhanced scattering immunoturbidimetry |
| ESR | Weil's method |
| Ionized calcium | colorimetric assay |
| PTH | Chemiluminescence immunoassay |
| 25(OH)VD | Chemiluminescence immunoassay |
| IL-6 | enzyme linked immunosorbent assay |
| IL-10 | enzyme linked immunosorbent assay |

**Abbreviations:** FPG: fasting plasma glucose; TCH: total cholesterol; TG: triacylglycerol; LDL-C: low-density lipoprotein cholesterol; HDL-C:high-density lipoprotein cholesterol; HbA1c: glycated hemoglobin A1c; CRP: C-reactive protein; ESR: erythrocyte sedimentation rate; PTH: parathyroid hormone; VD: vitamin D; IL-6: interleukin-6; IL-10: interleukin-10.
