# Supplementary material for: Level of 25-hydroxyvitamin D and vitamin D receptor in diabetic foot ulcer and factor associated with diabetic foot ulcers
Source: Diabetol Metab Syndr. 2023 Feb 24;15:30. doi: 10.1186/s13098-023-01002-3 (PMC9951493; doi:10.1186/s13098-023-01002-3)
Supplement: Supplementary file 3 — Additional file 3: Table S2. Specific primers and reaction conditions in qRT-PCR. [file 13098_2023_1002_MOESM3_ESM.doc]

**Table S2** Specific primers and reaction conditions in qRT-PCR

| Specific primers in qRT-PCR | |
| --- | --- |
| Target gene | Primer |
| VDR | forward, 5′-CTGACCCTGGAGACTTTGAC-3′ |
|  | reverse, 5′-TTCCTCTGCACTTCCTCATC-3′ |
| IL-6 | forward, 5′-GACAACTTTGGCATTGTGG-3′ |
|  | reverse, 5′-ATGCAGGGATGATGTTCTG-3′ |
| IL-10 | forward, 5′-AGAACCTGAAGACCCTCAGGC-3′ |
|  | reverse, 5′-CCACGGCCTTGCTCTTGTT-3′ |
| GAPDH | forward, 5′-GGAAGGTGAAGGTCGGAGTC-3′ |
|  | reverse, 5′-AATGAAGGGGTCATTGATGG-3′ |
| Reaction conditions in qRT-PCR | |
| 45 cycles of pre-denaturation at 95°C for 2 minutes, denaturation at 95°C for 15 seconds, annealing at 58°C for 30 seconds, and extending at 72°C for 10 seconds. | |

**Abbreviations:** qRT-PCR: quantitative real-time polymerase chain reaction; VDR: vitamin D receptor; IL-6: interleukin-6; IL-10: interleukin-10; GAPDH: glyceraldehyde-3-phosphate dehydrogenase.
