# Supplementary material for: Level of 25-hydroxyvitamin D and vitamin D receptor in diabetic foot ulcer and factor associated with diabetic foot ulcers
Source: Diabetol Metab Syndr. 2023 Feb 24;15:30. doi: 10.1186/s13098-023-01002-3 (PMC9951493; doi:10.1186/s13098-023-01002-3)
Supplement: Supplementary file 4 — Additional file 4: Table S3. Comparison of vitamin D nutritional status in diabetes foot ulcers with different Wagner grades and infection severity. [file 13098_2023_1002_MOESM4_ESM.doc]

**Table S3**  Comparison of vitamin D nutritional status in diabetes foot ulcers with different Wagner grades and infection severity [n (%)]

| Group | n | Vitamin D status n (%) | | | χ^2^ value | *P* value |
| --- | --- | --- | --- | --- | --- | --- |
|  |  | Deficiency | Insufficiency | Sufficiency |  |  |
| Wagner grade |  |  |  |  | 40.31 | <0.001 |
| II | 20 | 5 (25.0) | 9 (45.0) | 6 (30.0) |  |  |
| III | 108 | 83 (76.9) | 23 (21.3) | 2 (1.8) |  |  |
| IV | 28 | 24 (85.7) | 4 (14.3) | 0 (0.0) |  |  |
| Severity of infection |  |  |  |  | 23.86 | <0.001 |
| mild | 20 | 8 (40.0) | 7 (35.0) | 5 (25.0) |  |  |
| moderate | 94 | 69 (73.4) | 22 (23.4) | 3 (3.2) |  |  |
| severe | 42 | 35 (83.3) | 7 (16.7) | 0 (0.0) |  |  |

**Notes:** Data are presented numbers (%) among three groups analyzed using *x*^2^ test.

**Abbreviation:** DFU: diabetic foot ulcer.
